# Can Information and Entropic Dynamics Bridge the Gap Between Biology and the Physical Sciences?

**DOI:** 10.3390/e28030349

**Published:** 2026-03-20

**Authors:** Richard L. Summers, Melvin M. Vopson

**Affiliations:** 1 University of Mississippi Medical Center, Jackson, MS 39216, USA; 2 School of Mathematics & Physics, University of Portsmouth, Portsmouth PO1 2UP, UK; melvin.vopson@port.ac.uk

This Special Issue, entitled *Entropy and Information in Biological Systems*, comprises several unique contributions that attempt to bridge the gap between the fields of biology and the physical sciences. The methods used in these investigations are facilitated by our modern-day understanding of information and entropic dynamics [1,2,3,4,5,6,7,8]. The proposed use of entropy and information to advance our understanding of neuroscience in two of the featured articles highlights the importance of this area of study for both developing our understanding of cognition and also for the future of artificial intelligence [9,10]. Entropy and information dynamics are explored as driving genetic mechanisms in the featured studies of mutation and evolution [11,12], with such a perspective potentially reflecting a fundamental biological principle [13]. Another study takes a broad look at the role of information in the spatial patterns of biological systems [14]. Of particular interest is a study on the role of entropy and information for understanding the complexity of the clinical presentation of stroke in a practical systems analysis [15].

Until the 20th century, the physical and biological sciences were very distinct disciplines, with biology being highly dependent on qualitative descriptions of mechanisms with a somewhat teleonomic perspective. These differences were in part due to an inherent conflicting paradox in which physics describes the universe as moving toward an increase in entropy with maximum randomness, while life maintains local systems of order. In 1943, Erwin Schrödinger proposed that an understanding of the true nature of living systems first requires an apprehension of their ability to control entropy dynamics within their environment [16]. Subsequently, the development of information theory for communications by Claude Shannon was linked to the concept of entropy [17]. Living organisms utilize and exchange information as a form of biological currency during the process of maintaining their organizational structure and adapting to their environmental conditions. A deeper understanding of biological systems from the information/entropy perspective could provide considerable insights into the fundamental nature of their entropy dynamics and provide a foundation for a comprehensive theoretical biology [6,13].

Furthermore, a better comprehension of the role of information and entropic dynamics in biology could also provide greater insight into the machinations of the physical sciences [6,8], as, after all, biological systems are an integral part of the universe and must also obey the laws of physics. Understanding the complex mechanisms taking place in biological systems provides a unique feedback loop through which to elucidate physical processes that are inaccessible and non-testable in inorganic systems.

From the conception of entropy as a consequence of energy transitions, its qualifications, based on notions of certainty, order, predictability, usefulness, and knowability, have been naturally dependent on the inclusion of an observer. Uncertainty also implies a limitation in the capacity for discernment and prediction as determined through the lens of a living system. If the observer is to hold a central place in our modern comprehension of entropy, then it is important to incorporate biological principles into that description. Such an approach could broaden our understanding of physical entropic dynamics, including a determination of the meaning of information as a biological fundamental [18,19,20]. This Special Issue makes a small but important contribution in this regard. Moreover, with the emergence of Artificial Intelligence, new tools are becoming available to the scientific community that will likely see our understanding of physical entropic dynamics in physics and biology advance at an accelerated pace.

*“Observer participancy gives rise to information; and information gives rise to physics.”*

**—John Archibald Wheeler**

*“…general relativity, quantum mechanics, and statistical mechanics are actually derivable, and from the same ultimate foundation: the interplay between computational irreducibility and the computational boundedness of observers.”*

**—Stephen Wolfram**

*“But if the ultimate aim of the whole of Science is indeed, as I believe, to clarify man’s relationship to the Universe, then biology must be accorded a central position.”*

**—Jacques Monod**

*“It is the quality, not the mere existence, of information that is the real mystery here.”*

**—Paul Davies**
